# Another Case of Multilevel Cervical Disconnection Syndrome Presenting as Neonatal Encephalopathy

**DOI:** 10.1155/2018/7908753

**Published:** 2018-10-24

**Authors:** Kaylan M. Brady, Jonathan A. Blau, Spencer J. Serras, Jeremy T. Neuman, Richard Sidlow

**Affiliations:** ^1^Department of Pediatrics, Staten Island University Hospital-Northwell Health, Staten Island, NY, USA; ^2^Division of Neonatology, Department of Pediatrics, Staten Island University Hospital-Northwell Health, Staten Island, NY, USA; ^3^Division of Neuroradiology, Department of Radiology, Staten Island University Hospital-Northwell Health, Staten Island, NY, USA; ^4^Division of Pediatric Radiology, Department of Radiology, Staten Island University Hospital-Northwell Health, Staten Island, NY, USA; ^5^Division of Pediatric Hospitalist Medicine, Department of Pediatrics, Staten Island University Hospital-Northwell Health, Staten Island, NY, USA

## Abstract

Multilevel cervical disconnection syndrome (MCDS) is a rare malformation of the cervical spine previously documented in two toddlers. We present a case of a newborn first thought to have hypoxic-ischemic encephalopathy who was subsequently diagnosed with MCDS. The possibility of in utero presentation of the syndrome in this patient and the categorization of this syndrome in the spectrum of basilar skull/upper cervical malformation syndromes is discussed.

## 1. Introduction

Most congenital cervical spine anomalies are asymptomatic and, if ever, present well after birth or are found incidentally on radiographic imaging. These known anomalies, including basilar impression, occipitocervical synostosis, odontoid anomalies, and Klippel-Feil syndrome, can present with neck pain, weakness, and upper extremity numbness. These anomalies, however, are not known to cause symptoms at birth, in the neonatal period, or even in infancy.

Two childhood cases of symptomatic cervical spine anomalies have been reported, both distinct from other known anomalies, in which the authors coined the term “multilevel cervical disconnection syndrome (MCDS)” [1]. Both cases presented due to clinical symptoms of spinal cord compression as toddlers. Neither of these cases, however, presented with symptoms at birth.

We present below a case of a baby boy born with MCDS causing spinal cord compression and encephalopathy at birth.

## 2. Case Description

A baby boy was born at 38 weeks and six days gestation to a primigravid woman. The mother was followed by a high-risk obstetrician due to SSRI use during the initial months of pregnancy. Otherwise, the maternal history was unremarkable, and the prenatal history was significant only for breech presentation. The mother presented to labor and delivery due to a four-week history of decreased fetal movements. A nonstress test was performed which was reactive with good fetal heart rate; however, a biophysical profile performed immediately afterwards was 6/10 due to lack of fetal movement/fetal tone. The mother was admitted for an emergent cesarean section delivery.

Once delivered, the baby was floppy, apneic, pallid, and bradycardic. Neonatal Resuscitation Protocol was initiated and after no response to tactile stimulation and positive pressure ventilation (PPV), a Code 100 was called. Chest compressions were begun and the baby was intubated. Apgar scores were two at one minute and five at five minutes, respectively. Two doses of epinephrine were given via the endotracheal tube (ET) and an umbilical venous catheter was placed emergently. A normal saline bolus was given due to poor perfusion, pallor and bradycardia. The Apgar score at ten minutes was seven. The baby was then given PPV through the ET tube during transportation to the neonatal intensive care unit (NICU).

Upon arrival to the NICU, the baby was flaccid, hypothermic, bradycardic, apneic, and hypoxic. The baby had no spontaneous respirations. The neurological examination revealed absent cry, Moro, gag, suck, swallow and grasp reflexes, and decreased tone throughout with intermittent twitching of the left upper and lower extremities. The spine was grossly normal. Arterial blood gases on admission revealed a mixed respiratory and metabolic acidosis (pH 6.92 (nl 7.38-7.42)/pCO2 78 mmHg (nl 38-42 mmHg)/pO2 92 mmHg (nl 94-98 mmHg)/HCO3 16.2 mEq/L (nl 23-27 meq/L)/ BE-16.4 (nl -2-2)). Since the physical examination findings and presentation were consistent with hypoxic-ischemic encephalopathy (HIE), a 72-hour neonatal whole body cooling protocol was begun at hour 6 of life [2]. The baby was also started on ampicillin and gentamicin for presumed sepsis, which were discontinued after 48 hours once the blood culture result was negative.

While being cooled, the baby began to have inconsistent symmetric spontaneous movements of his extremities, withdrawal of all extremities, and symmetric facial movements in response to painful stimuli. There was no spontaneous eye opening, but he resisted eye opening. Patellar and bicep reflexes were I/IV and toes were down going. Video electroencephalogram revealed mild slowing initially, but normalized by day three of life with no slowing or epileptiform activity. The baby remained ventilator dependent and failed multiple apnea tests.

On day of four of life, the baby was rewarmed with improvement in overall tone, a now inconsistent gag reflex, strong withdrawal to painful stimuli, and II/IV reflexes throughout. Despite the baby's slightly improved neurological exam, he remained ventilator dependent with no spontaneous respirations.

**Figure 1:**
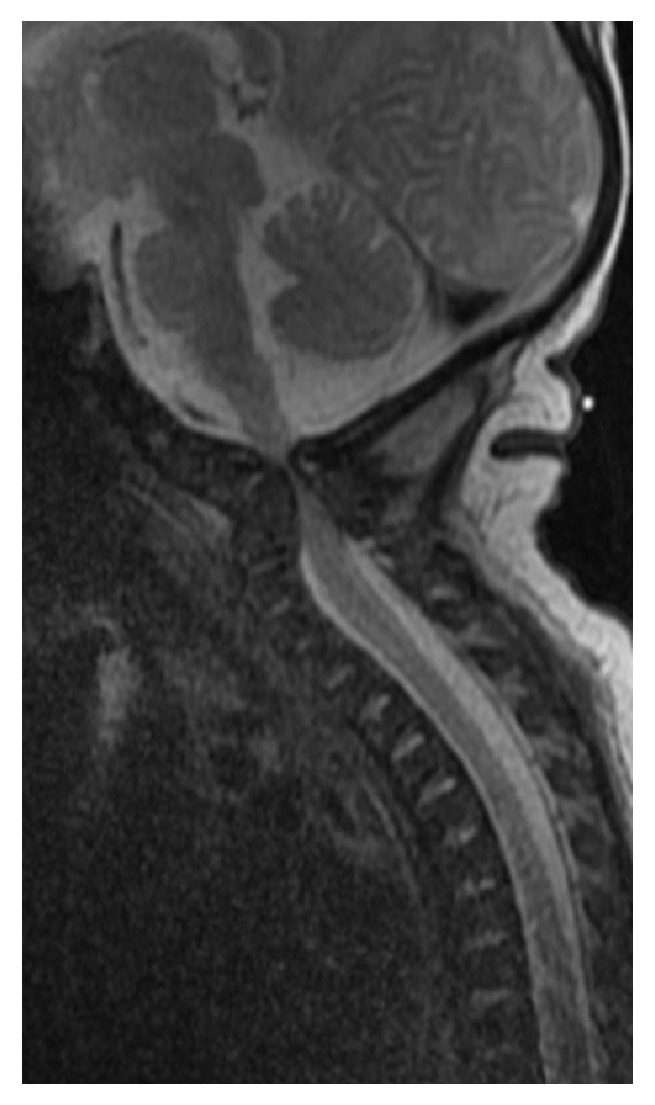
MRI of cervical spine: spinal cord compression at level of the foramen magnum secondary to craniocervical junction anomaly with focal severe kyphosis of the upper cervical spine (C3-C4).

**Figure 2:**
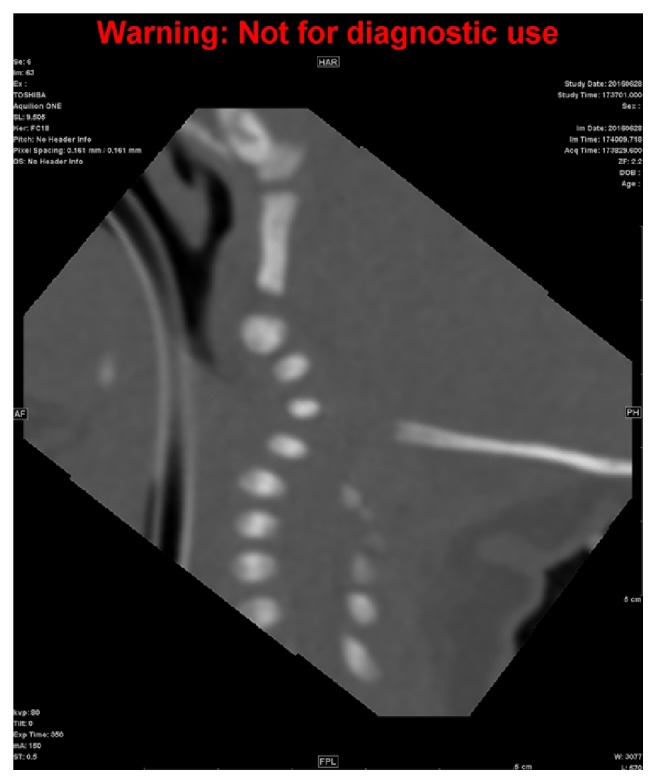
CT of cervical spine: occipitalization/assimilation of the atlas with severe kyphoscoliosis of the upper cervical spine, resulting in severe spinal canal stenosis with spinal cord compression. Incomplete/absent ossification centers of the upper cervical spine.

**Figure 3:**
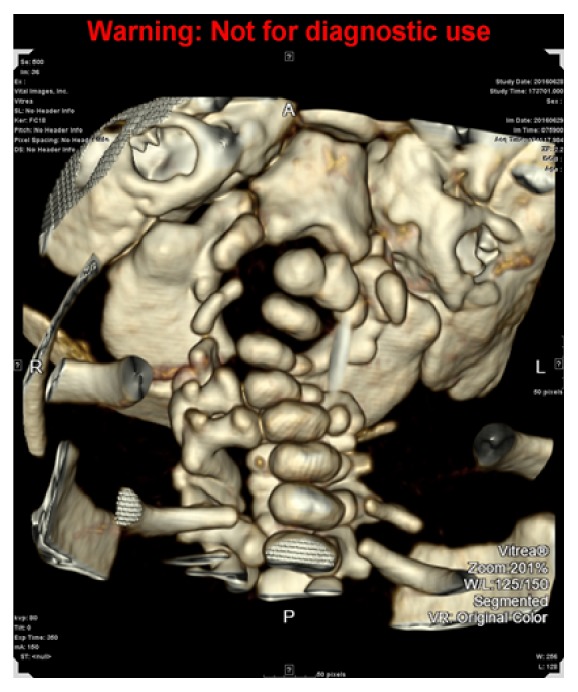
3D image of MRI of cervical spine: absent pedicle in the midcervical spine.

Magnetic resonance imaging (MRI) of the brain performed on day five of life revealed no definitive evidence of acute infarct or hypoxic/anoxic injury, but revealed an abnormality of the cervical spine. MRI of the cervical spine revealed spinal cord compression at the level of the foramen magnum secondary to a craniocervical junction anomaly with severe kyphosis of the upper cervical spine at the level of C3-C4 (Figure 1). Computerized tomography of the cervical spine confirmed the diagnosis along with occipitalization and assimilation of the atlas and absent ossifications centers of the upper cervical spine (Figures 2 and 3).

Upon the diagnosis of spinal cord compression, the baby was transferred for urgent neurosurgical evaluation and treatment. Despite surgical intervention with suboccipital craniotomy and posterior cervical laminectomy with rib graft spinal fusion, no improvement was noted and the baby had no purposeful movements postoperatively. He later underwent tracheostomy for persistent respiratory failure. The patient was discharged to a specialty rehabilitation center where he remained for nine months and was subsequently discharged home. The patient has remained quadriplegic and ventilator dependent without any interval improvement in motor or respiratory function.

## 3. Discussion

The incidence of congenital anomalies of the cervical spine is much less common than HIE, the former occurring in approximately 1 in 40,000 to 42,000 births versus 1.5 per 1,000 live births in the United States [3, 4]. Basilar impression, occipitocervical synostosis, odontoid anomalies, and Klippel-Feil syndrome do not present symptomatically at birth and are not known to cause severe spinal cord compression requiring mechanical ventilation [5]. Therefore, cervical spine anomalies and spinal cord compression were not in the differential diagnosis of this baby at the time of presentation or during his initial clinical course. Retrospectively, the encephalopathic presentation of this baby can be explained by the combination of phrenic nerve palsy and the neurological sequelae resulting from high cervical spinal cord compression. Additionally, given the absence on physical examination of other facial, orthopedic, and cardiac findings, no syndromic explanation (e.g., Larsen Syndrome) was considered.

Two childhood cases with similar radiographic findings have been reported by Klimo et al., one in a 22-month-old female with myelopathy and a neck deformity and another in a three-year-old male with upper and lower extremity weakness. Both patients were previously healthy and had no significant birth history. Radiographic imaging of the cervical spine in both cases showed multilevel abnormalities of the cervical pedicles, cervical kyphosis, and spinal cord compression. The authors termed this condition “multilevel cervical disconnection syndrome (MCDS)”, due to the apparent disconnect between the anterior and posterior column. Both cases were repaired surgically in a similar fashion with improvement of symptoms postoperatively. These two cases, however, presented as toddlers, were normal at birth and had a much less severe component of cervical kyphosis and spinal cord compression [1].

The above authors believed that the embryological cause of the abnormalities in the cervical spine was due to a defect in ossification and chondrification at the level of the cervical pedicles [1]. Others have theorized that Chiari-1 malformation, cervical spina bifida occulta, cervical scoliosis, Klippel-Feil deformity, atlantoaxial assimilation, atlantooccipital fusion, and basilar invagination may be developmental variants along a spectrum of severity common to the base of the skull and upper cervical spine [6]. In patients without collagenopathies, mutations in the genes GDF3 and GDF6 which code for osseous growth differentiation factors, defects of postotic neural crest (PONC) cells, or subclavian artery supply disruption sequence (SASDS) have been proposed as explanations for the anomalies previously listed [7–9]. We would add (MCDS) to this list and invoke these same explanations as possible etiologies of this syndrome.

Our case had similar radiographic findings with at least one missing pedicle at the level of the midcervical spine along with obvious cervical kyphosis and incomplete/absent ossification centers (Figure 3). The profound in utero presentation of this baby was likely caused by the apex of cervical kyphosis being at the level of C4 compressing the spinal cord down to approximately one millimeter in diameter early on in development.

This is the first case of MCDS documented in a neonate and with symptoms documented in utero. Spinal cord compression due to cervical spine anomalies, although rare, should therefore be kept in the differential diagnosis of a newborn suspected of having neonatal encephalopathy, as it can present with respiratory failure due to phrenic nerve palsy with neurological sequelae. Additional imaging of the cervical spine is needed for diagnosis and can be performed concomitantly with the brain imaging necessary to confirm HIE. This also expands the differential diagnosis to include additional structural abnormalities other than vertebral bony anomalies at the level of the cervical spine that could cause spinal cord compression and phrenic nerve palsy, such as an arteriovenous malformation (AVM). One case has been documented in a full-term baby born with flaccid quadriparesis that remained ventilator dependent, found on MRI to have an epidural hematoma compressing the cervical cord, later attributed to an AVM on postpartum examination [10].

## 4. Conclusion

Although extremely rare, spinal cord compression due to cervical spine anomalies should be kept in the differential diagnosis of a baby presenting with encephalopathy, as it can cause phrenic nerve palsy resulting in respiratory failure and neurological sequelae. Radiologic imaging can be performed early in the clinical course in conjunction with the ongoing workup for neonatal encephalopathy. This can allow for a more rapid diagnosis and treatment plan and ultimately confirm or eliminate a potential cause for the neonate's symptoms.

## References

[1] Klimo P., Anderson R. C. E., Brockmeyer D. L. (2006). Multilevel cervical disconnection syndrome: Initial description, embryogenesis, and management: Report of two cases. *Journal of Neurosurgery*.

[2] Shankaran S., Laptook A. R., Ehrenkranz R. A. (2005). Whole-body hypothermia for neonates with hypoxic-ischemic encephalopathy. *The New England Journal of Medicine*.

[3] Klimo P., Rao G., Brockmeyer D. (2007). Congenital Anomalies of the Cervical Spine. *Neurosurgery Clinics of North America*.

[4] Kurinczuk J. J., White-Koning M., Badawi N. (2010). Epidemiology of neonatal encephalopathy and hypoxic-ischaemic encephalopathy. *Early Human Development*.

[5] Medscape Medscape: Congenital spinal deformity. New York, USA, 2017. https://emedicine.medscape.com/article/1260442-overview#a6.

[6] Wong S.-L., Paviour D. C., Clifford-Jones R. E. (2008). Chiari-1 malformation and the neck-tongue syndrome: Cause or coincidence?. *Cephalalgia*.

[7] Bavinck J. N. B., Weaver D. D. (1986). Subclavian artery supply disruption sequence: hypothesis of a vascular etiology for Poland, Klippel-Feil, and Möbius anomalies. *American Journal of Medical Genetics*.

[8] Markunas C. A., Soldano K., Dunlap K. (2013). Stratified whole genome linkage analysis of chiari type i malformation implicates known klippel-feil syndrome genes as putative disease candidates. *PLoS ONE*.

[9] Matsuoka T., Ahlberg P. E., Kessaris N. (2005). Neural crest origins of the neck and shoulder. *Nature*.

[10] Coulter D. M., Zhou H., Rorke-Adams L. B. (2007). Catastrophic intrauterine spinal cord injury caused by an arteriovenous malformation. *Journal of Perinatology*.

